# Reconciling Simulations and Experiments With BICePs: A Review

**DOI:** 10.3389/fmolb.2021.661520

**Published:** 2021-05-11

**Authors:** Vincent A. Voelz, Yunhui Ge, Robert M. Raddi

**Affiliations:** ^1^Department of Chemistry, Temple University, Philadelphia, PA, United States; ^2^Department of Pharmaceutical Sciences, University of California, Irvine, Irvine, CA, United States

**Keywords:** Bayesian inference, conformational populations, MCMC, cyclic peptides, peptoids, peptidomimetics, HDX protection factors, molecular simulation

## Abstract

Bayesian Inference of Conformational Populations (BICePs) is an algorithm developed to reconcile simulated ensembles with sparse experimental measurements. The Bayesian framework of BICePs enables population reweighting as a post-simulation processing step, with several advantages over existing methods, including the proper use of reference potentials, and the estimation of a Bayes factor-like quantity called the BICePs score for model selection. Here, we summarize the theory underlying this method in context with related algorithms, review the history of BICePs applications to date, and discuss current shortcomings along with future plans for improvement.

## 1. Introduction

Bayesian Inference of Conformational Populations (BICePs) is an algorithm developed to reconcile simulated ensembles with sparse experimental measurements. The inputs to BICePs are: (1) a set of discrete conformational states and corresponding populations predicted from a theoretical prior, and (2) a set of experimental observables. The primary output of BICePs are estimates of reweighted conformational populations that balances the information from theory and experiment using a Bayesian framework.

The Bayesian Inference of Conformational Populations (BICePs) algorithm arose from a need to predict conformational ensembles of organic molecules with significant structural heterogeneity in solution, such as natural product macrocycles and peptidomimetics. Specifically, we aimed to develop an approach that leaned more heavily on high-quality theory/simulation-based conformational ensembles, to be later reconciled with potentially sparse experimental observables.

Existing methods for this purpose, such as NAMFIS (NMR Analysis of Molecular Flexibility in Solution, Cicero et al., 1995) and DISCON (Distribution of in-solution conformations, Atasoylu et al., 2010) were used primarily by the organic chemistry community in the context of NMR refinement. While these methods do estimate populations of conformational states, they are essentially a kind of “maximum parsimony” method, where all possible solution-state conformations are enumerated in order to find a small number of structures compatible with NMR-based constraints. Such approaches are less useful for simulated structural ensembles, for which ensemble-averaged observables should be restrained, in a way that can sufficiently account for uncertainties in experimental measurements.

Another class of algorithms, categorized as “maximum entropy” approaches (Pitera and Chodera, 2012; Bonomi et al., 2017; Orioli et al., 2020) focus primarily on using bias potentials to enforce constraints on an experimental observable throughout the course of a molecular simulation. While this can be approximated efficiently in practice by restraining replica-averaged observables (Vendruscolo et al., 2003; Best and Vendruscolo, 2006) it must be modified to account for experimental uncertainty, a problem more recently addressed by the Metainference algorithm of Bonomi and Vendruscolo (Bonomi et al., 2016a,b) which employs Bayesian inference.

In contrast to this approach, we sought a method that could reweight a *discrete* set of conformational populations as a “post-processing” step, after a simulation was performed. Such *post-hoc* reweighting would nicely complement Markov State Model approaches for biomolecular simulation, which require partitioning of trajectory data into discrete conformational states. Another reason to develop a reweighting approach was the growing problem of bespoke force field parameterization for peptidomimetics. A reweighting approach could enable a sufficiently accurate general force field [e.g., GAFF Wang et al., 2004] to generate an initial model of conformational populations that could then be further refined against experimental data.

BICePs was modeled closely after the Inferential Structural Determination (ISD) algorithm of Rieping, Habeck, and Nilges (Rieping et al., 2005). Like BICePs, ISD is a Bayesian approach where simulated conformational populations are used as the Bayesian prior, and experimental restraints form the likelihood function. The full posterior distribution of conformational states and model parameters is then sampled using a Monte Carlo algorithm (Habeck et al., 2006). But as we soon discovered when developing BICePs, not all experimental restraints impart the same amount of information, and BICePs makes critical improvements over ISD by accounting for this fact.

The information gained upon obtaining a measurement is relative to the prior information we possess. For example, suppose we want to use Bayesian inference to refine the conformational distribution of a linear peptide, given an experimental distance measurement between two residues. The measurement is highly informative if the the residues are distant in sequence, but non-informative if the residues are close in sequence. To account for a more diverse range of informative experimental restraints, BICePs implements *reference potentials*, which are discussed more fully in the Theory section.

As a consequence of better accounting for the information content of experimental restraints, BICePs is able to calculate a Bayes factor-like quantity, that we call the *BICePs score*, that can be used for model selection. The BICePs score is highly useful: it is a number that can report the extent to which a conformational ensemble is consistent with experimental data. Not only can this be used to show that reweighted populations are more consistent with experimental data, it can also be used to rank different simulated ensembles by their accuracy in reproducing experimental observables (Ge and Voelz, 2018). While there are still some improvements to BICePs needed to use this for automated force field validation (see Discussion), the BICePs score is highly useful, and we expect it will continue to provide attractive incentive to use this algorithm.

In the remainder of this review, we will first discuss the theory underlying the BICePs algorithm, and describe some of the ways we implement this theory to sample the Bayesian posterior distribution of conformational state and model parameters. We then provide a case-by-case review of past examples where BICePs has been applied to model conformational distributions. Finally, we discuss some of the shortcomings of BICePs and ongoing challenges we hope to address with future improvements to BICePs.

## 2. Theory

### 2.1. Bayesian Inference

The goal of BICePs is to model a *posterior* distribution *P*(*X*|*D*) of conformational states *X*, given some experimental data *D*. This posterior probability is proportional to a product of (1) a *likelihood* function *Q*(*D*|*X*) representing experimental restraints, and (2) a *prior* distribution *P*(*X*).

(1) P(X|D)∝Q(D|X)P(X)

The prior distribution, *P*(*X*), represents prior knowledge about the populations of conformational states *X* derived from theoretical modeling. This distribution can computed directly from a molecular simulation, or come from any number of theoretical models of the conformational free energy landscape (e.g., from QM calculations).

The likelihood function, *Q*(*D*|*X*), reflects how well a given conformation *X* agrees with experimental measurements. It is assumed to obey a normally-distributed error model of the form:

(2) $$Q(D|X,\sigma )=\prod_{j}\frac{1}{\sqrt{2\pi \sigma^{2}}}\exp\left(-{\left[r_{j}(X)-r_{j}^{\exp}\right]}^{2}/2\sigma^{2}\right).$$

Here, the data *D* comprise a set of experimental observables indexed by *j* = 1, ..., *N*_*j*_. The *r*_*j*_(*X*) represent observables back-calculated from the theoretical model (ensemble-averaged over states within *X*), and $r_{j}^{\exp}$ represent the experimental values of each observable. In Equation (2), we assume that each experimental observable has the same uncertainty σ. In practice, different types of observables *r*_*j*_ can be assigned specific uncertainties σ_*j*_, although this is usually done in groups (different values of σ_*j*_ for sets of NOE distances, J-coupling constants, etc.) for the sake of computational efficiency. There are of course many situations where experimental uncertainty can vary even within different sets measurements, which can be addressed by defining custom restraint groups.

The likelihood function *Q*(*D*|*X*) can be thought of as the quantity that reweights the prior estimate of the population *P*(*X*). Conformational states *X* that better agree with the experimental measurements get higher weights. An important distinction to note: as BICePs is currently formulated, the likelihood function *Q*(*D*|*X*) compares the experimental value $r_{j}^{\exp}$ to the back-calculated observable *r*_*j*_(*X*) of a *single* conformational state *X*, rather than an ensemble-averaged back-calculated observable $\langle r_{j}\rangle =\underset{X}{\sum }r_{j}\left(X\right)P\left(X\right)$. Consequently, the error model parameter σ reflects both uncertainty in the experimental measurements and heterogeneity in the conformational ensemble. Errors in the forward model *r*_*j*_(*X*) are included in σ, and in many cases may dominate the experimental errors (chemical shifts being the most dramatic example).

As for choosing values of the uncertainty parameter(s) σ, these uncertainties are usually not known *a priori*, and must be treated as a so-called *nuisance parameter*, which can be modeled using some prior model *P*(σ):

(3) P(X,σ|D)∝Q(D|X,σ)P(X)P(σ)

While we don't know the exact value of σ, we treat *P*(σ) as non-informative Jeffreys prior [*P*(σ) ~ 1/σ], and include this parameter in the posterior in order to sample the joint distribution of (*X*, σ). Then *P*(*X*|*D*) can then be obtained as the marginal distribution ∫*P*(*X*, σ|*D*)*dσ*. In the case where estimates of the errors from experiments *are* known, a limited range of possible σ values can be imposed.

Because the likelihood function enforces restraints on individual conformational states (not ensemble-averages), *P*(*X*|*D*) represents an “uncertainty ensemble” rather than a “statistical ensemble” of conformational populations, to use the language of Bonomi et al. (2017). However, it is quite useful think of *P*(*X*|*D*) as conformational populations, as we show in the examples below. For example, if *P*(*X*|*D*) gives equal values for two conformational states, then BICePs predicts they are equally consistent with the experimental data. While BICePs doesn't explicitly predict a mixture of two conformations, by maximum ignorance (i.e., MaxEnt) we would infer equal populations in the conformational ensemble. Future improvements to BICePs will address this by constraining ensemble averages across multiple replicas (see Discussion).

### 2.2. Reference Potentials

While the likelihood function *Q*(*D*|*X*) weights the conformational space *X*, the actual restraints exist in some restraint space **r**, a low-dimensional projection of the state space of *X*. As a result, we need to introduce a reference potential *Q*_ref_(**r**) that reflects the distribution of observables **r** in the *absence* of any experimental measurements. With this modification, Equation (1) becomes:

(4) $$P(X|D)\propto \left[\frac{Q(r(X)|D)}{Q_{\text{ ref }}(r(X))}\right]P(X).$$

The negative logarithm of the bracketed weighting function, −ln [*Q*(**r**|*D*)/*Q*_ref_(**r**)], can be thought of as equivalent to a potential of mean force (Hamelryck et al., 2010; Olsson et al., 2011, 2013). With a proper reference potential, the relative information content of each restraint becomes meaningful. In our previous work, we have shown that using BICePs with proper reference potentials can be essential for obtaining accurate results (Voelz and Zhou, 2014; Ge and Voelz, 2018).

As an example of why reference potentials are needed, consider experimental measurements of interresidue distances in a protein. A distance measurement of 5 Å for a pair of residue indices (*i*, *i* + 2) is essentially non-informative, since we already know these residues are close in sequence along the polypeptide chain, while a distance measurement of 5 Å for (*i*, *i* + 50) is highly informative. The ratio *Q*(**r**(*X*)|*D*)/*Q*_ref_(**r**(*X*)) is needed to correctly characterize the change in our state of knowledge.

The choice of what reference potential to use in a particular situation is subject to some interpretation. Since BICePs is designed to be used with sparse and/or noisy experimental data, the likelihood function *Q*(*D*|*X*) typically enforces experimental restraints smoothly over broad ranges of values. Similarly, reference potentials should be sufficiently smooth and broad, to avoid regions of restraint space with unrealistically small values of *Q*_ref_(**r**), which may in turn produce artificially inflated weights for certain conformations. For this reason, we advocate the use of conservative reference potentials, which do not make unnecessary assumptions about the underlying distribution of a given observable in the absence of experimental information.

We currently support three kinds of reference potentials in our software implementation of BICePs: (1) uniform (non-informative), (2) exponential, and (3) Gaussian. An exponential reference potential is the least-informative distribution if only the first moment of *Q*_ref_ (the mean, 〈*r*〉) is known. A Gaussian distribution is the least-informative distribution if only the first and second moments are known (〈*r*〉 and 〈*r*^2^〉).

As an interesting example, consider the reference potential used for a set of interproton distances measured in an NMR study of a 14-membered macrocycle, a situation we considered in Voelz and Zhou (2014). In the absence of all other information, our reference information is that the space of molecular conformations are 14-membered rings. At the very least, the the distribution of interproton distances must be non-negative, and bounded from above. To get an idea of the empirical distribution of possible interproton distances, we examined all input conformations to BICePs, regardless of their energy, and found no clear pattern other than a well-defined mean. Therefore, we chose an exponential function as the reference potential. In practice, the reference potential was fairly flat, since the average interproton distance had a mean near 4 Å, and a maximum near 5 Å.

### 2.3. Sampling the Posterior Using MCMC

Markov Chain Monte Carlo (MCMC) is used to sample the posterior distribution of *X* and σ, with −ln *P*(*X*, σ|*D*) used an the effective energy function. The energy function can be obtained as the negative logarithm of the posterior probability given in Equation (3):

(5) $$\begin{array}{l} -\ln P(X,\sigma |D)=\left(N_{j}+1\right)\ln\sigma +\chi^{2}(X)/2\sigma^{2}-\ln Q_{\text{ref}}\\ \;+\left(N_{j}/2\right)\ln2\pi -\ln P(X). \end{array}$$

The quantity χ^2^(*X*) is the sum of squared errors, computed as

(6) $$\chi^{2}(X)=\sum_{j}w_{j}{\left(r_{j}(X)-r_{j}^{\exp}\right)}^{2}$$

where *w*_*j*_ is a weight parameter designated for equivalent observables (For example: *w*_*j*_ = 1/3 is used for hydrogens in a methyl group).

**Figure 1:**
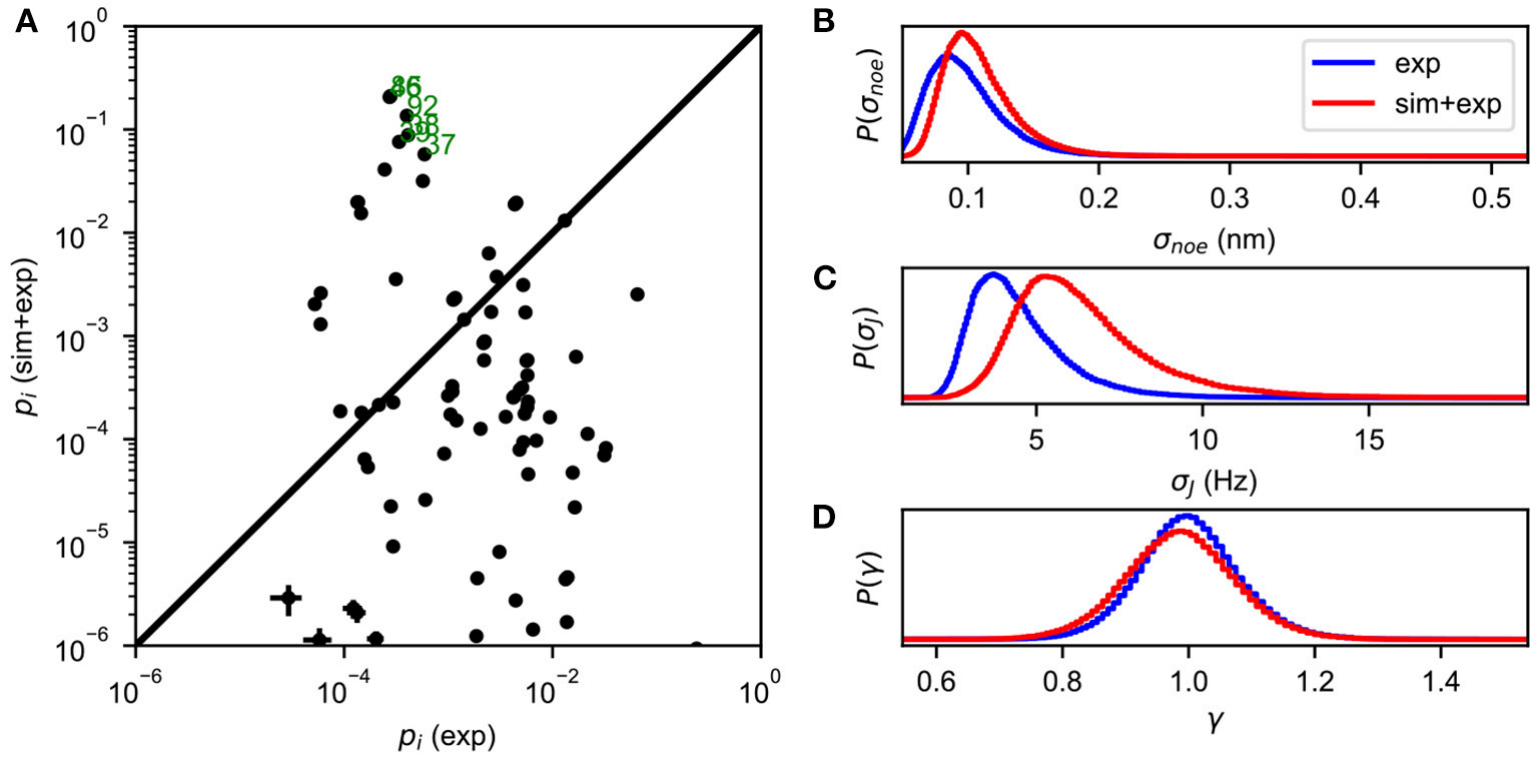
An example of BICePs output for albocycline (Liang et al., 2018). **(A)** A comparison of conformational state populations *p*_*i*_ (exp) inferred using only experimental restraints, vs. BICePs populations *p*_*i*_ (sim + exp) inferred using a combination of the simulation-based prior and experimental restraints. States on the lower right are highly compatible with experimental restraints, but are conformationally strained according the simulation model. Conformational states near the top of the graph are both reasonably compatible with experimental restraints, and highly-populated according to the simulation model. States labeled in green correspond closely to the two crystal isoforms of albocycline. **(B)** The marginal posterior distribution of σ_noe_, the uncertainty parameter for NOE distance restraints. **(C)** The marginal posterior distribution of σ_*J*_, the uncertainty parameter for *J*-coupling constants. **(D)** The marginal posterior distribution of γ, the scaling parameter for the NOE distances, remains near 1.0 throughout the MCMC sampling.

The Metropolis-Hastings algorithm is used to perform MCMC sampling of the energy function defined in Equation (5), yielding an estimate of the full posterior distribution *P*(*X*, σ|*D*). The most probable values of σ can be obtained by the marginal distribution *P*(σ|*D*) = ∫*P*(*X*, σ|*D*)*dX*, and the state populations are estimated as *P*(*X*|*D*) = ∫*P*(*X*, σ|*D*)*dσ* (Figure 1).

#### 2.3.1. Enhanced Sampling of the Posterior

Accurate BICePs results require sufficiently converged sampling of the entire (*X*, σ) landscape. To achieve enhanced sampling of *P*(*X*, σ|*D*), we use a free energy perturbation (FEP) method, where posterior sampling for a series of models with priors $P_{\lambda }\left(X\right)\sim {\left[P\left(X\right)\right]}^{\lambda }$, where 0 ≤ λ ≤ 1. The λ value serves to linearly scale the −ln *P*(*X*) term in Equation (5). The expanded ensemble of posterior distributions *P*_λ_(*X*, σ|*D*) thus spans a range of prior information: When λ = 0, the prior *P*_λ_(*X*) prior is uniform, and there is no information from theoretical modeling included in the sampling. When λ = 1, all the information from theoretical modeling is included in the sampling.

In the current implementation of BICePs, MCMC is performed in parallel for a fixed number of λ values ranging from 0 to 1. The multistate Bennett acceptance ratio (MBAR) free energy estimator (Shirts and Chodera, 2008) is then used to integrate samples from each ensemble to make statistically optimal estimates of all *P*_λ_(*X*|*D*).

### 2.4. The BICePs Score

The quality of a model *k* that uses a prior *P*^(*k*)^(*X*) from theoretical modeling can be assessed by the posterior likelihood *Z*^(*k*)^ of model *k*:

(7) $$Z^{\left(k\right)}=\int P^{\left(k\right)}(X,\sigma |D)dXd\sigma =\int P^{\left(k\right)}(X)Q(X)dX.$$

One way to think of *Z*^(*k*)^ is as an integral over the entire input space (including nuisance parameters) of the model. Another way, however, is to think of *Z*^(*k*)^ as an *overlap* integral between the prior *P*^(*k*)^(*X*) and a likelihood function *Q*(*X*) = ∫[*Q*(**r**(*X*)|*D*, σ)/*Q*_ref_(**r**(*X*))]*P*(σ)*dσ*. This integral reaches the maximum when *P*^(*k*)^(*X*) most closely matches the likelihood distribution *Q*(*X*) specified by the experimental restraints.

Suppose we have two models (1) and (2) with priors *P*^(1)^ and *P*^(2)^, and we want to know which one is more consistent with experimental measurements. In Bayesian statistics, the comparison is often made using the ratio of posterior model probabilities, *Z*^(1)^/*Z*^(2)^, also called the Bayes factor.

In BICePs, we consider a free energy-like quantity, called the BICePs score:

(8) $$f^{\left(k\right)}=-\ln\frac{Z^{\left(k\right)}}{Z_{0}},$$

which compares a model probability *Z*^(*k*)^ to a standard reference *Z*_0_ where no theoretical information is used (i.e., a model using the prior *P*_λ_(*X*) where λ = 0). The use of this standard reference is useful in several ways. For one, if the BICePs score *f*^(*k*)^ is *positive* for a given model *k*, it means that the theoretical model is *worse* than a totally uninformative prior–the theoretical model is somehow inconsistent with experiment. More importantly, since the BICePs score *f*^(*k*)^ is always computed against an absolute reference, it is a scalar quantity that can be used to perform model selection. The BICePs score therefore can be very useful for automatic model selection; for example, molecular simulation force field validation and parameterization (Ge and Voelz, 2018).

Unlike maximum-likelihood approaches, The BICePs score has the advantage of avoiding overfitting to a particular set of experimental observable values, especially when the data are sparse and/or noisy. Consider an alternative approach where the values of σ_*j*_ that maximize the posterior are identified for two different models and used to compute χ^2^ values for model selection. The χ^2^ values only compare the models at particular points in parameter space, while the BICePs score considers the total evidence integrated over all of parameter space.

## 3. Applications of BICePs

**Figure 2:**
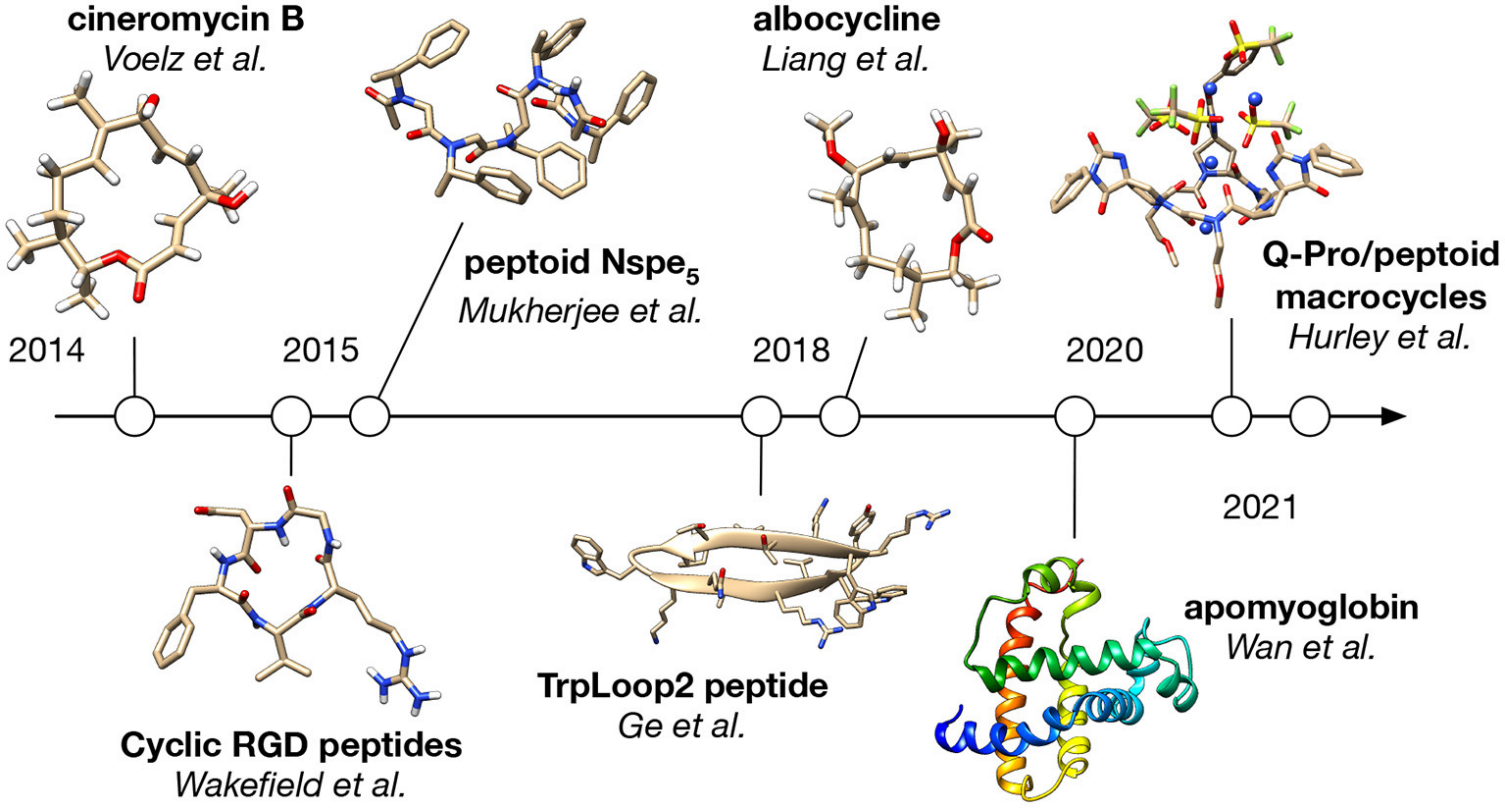
A timeline of BICePs application.

Applications of BICePs to date fall into two main categories: studies of small molecules like peptides and peptidomimetics, and studies of larger proteins like apomyoglobin (Figure 2).

### 3.1. Modeling Macrolide Antibiotics

The first application of BICePs, described in the seminal article that first introduced the algorithm, was to determine the solution-state conformational populations of the 14-membered macrolide antibiotic cineromycin B (Voelz and Zhou, 2014). Knowledge of solution-state structure is essential to identify potential targets of natural products, and to rationally design new kinds macrolide antibiotics.

A combination of theoretical modeling and sparse experimental NMR observables were used as input to BICePs. The theoretical modeling was performed in two steps. First, implicit solvent replica-exchange molecular dynamics (REMD) simulation in GAFF was performed to exhaustively sample the conformational landscape. The resulting sampling was then clustered into 100 discrete states. Next, each cluster center was subjected to QM minimization and single-point energy calculation at the B3LYP/6-3111G(2d,p)//HF/6-31G(d) level of theory. State populations were considered to be Boltzmann-distributed according to the computed QM energies. The sparse experimental constraints consisted of 13 interproton NOEs and five ${\text{vicinal}}^{3}J_{\text{HH}}$ coupling constants.

For this system, BICePs predicted a nearly equal mixture of two main conformational populations, each closely similar in structure to the two crystal isoforms found for albocycline, the O-methylated analog of cineromycin B. This work also showed the importance of the reference potentials in producing more correct posterior models.

In subsequent work, BICePs predicted a similar (nearly equal) mixture of solution-state populations for albocycline, using 12 NOE distance restraints and seven dihedral restraints from ${\text{vicinal}}^{3}J_{\text{HH}}$ coupling constants (Chatare and Andrade, 2017). This information helped inform molecular simulation and computational docking studies of albocycline binding to MurA, an enzyme involved in peptidoglycan biosynthesis, a potential new target for Methicillin-resistant *Staphylococcus aureus* (MRSA) infection (Liang et al., 2018).

### 3.2. Modeling Peptoid Foldamers

Peptoids (N-substituted oligoglycines) are a class of sequence-specific peptidomimetics that can be easily synthesized, and fold into unique structural scaffolds (Sun and Zuckermann, 2013). While the peptoid backbone is achiral and lacks hydrogen bond donors, rational design of N-substituents can control the amide *cis*/*trans* populations and secondary structure. An important goal for molecular modeling and simulation of these systems is to accurately predict solution-state conformational populations. Reliable methods to do this would enable the computational design of preorganized peptoid structural scaffolds to function as new bio-inspired materials or therapeutics (Voelz et al., 2011; Butterfoss et al., 2012; Kang et al., 2017; Schneider et al., 2018; Gimenez et al., 2019).

A particular challenge in simulating peptoids is the lack of accurate force fields. Unlike peptides, the chemical diversity of N-substituents is practically limitless, with each new peptoid residue requiring custom parameterization. BICePs can help avoid this by using a general-purpose force field to generate a prior conformational distribution, to be further refined against experimental data.

An example of this approach was pursued by Mukherjee et al. to model the solution-state conformational populations of an (S)-*N*-(1-phenylethyl) glycine pentamer, (Nspe)_5_, whose bulky chiral N-subtituents help this sequence fold into a right-handed *cis*-amide helix (Mukherjee et al., 2015). Disagreement between *ab initio* dihedral scans of the Nspe residue and the results of GAFF simulations motivated the development of an improved backbone potential, GAFF-ϕ, to better model the right-handed (negative ϕ-angle) preference of Nspe oligomers in solution.

BICePs was used to reweight GAFF and GAFF-ϕ predictions using sparse experimental restraints derived from previously published NMR studies: NOE distances (Armand et al., 1998) and *cis*/*trans* amide equilibria (*K*_ct_ ~ 2.5). BICePs scores for both GAFF and GAFF-ϕ were negative, suggesting the models are compatible with experiment. However, the GAFF-ϕ model was found to have a likelihood of 1.5 times that of the GAFF model, indicating it to be superior. Indeed, GAFF-ϕ predicted a much higher *cis*-amide helix population for (Nspe)_5_, consistent with previous NMR refinement and circular dichroism measurements.

By reweighting pre-defined conformational states, BICePs also provides a convenient methodology to avoid costly sampling. Unlike peptides, peptoids can populate both *cis* and *trans* amide conformations. Amides have large isomerization barriers in most force fields, typically requiring enhanced sampling methods like REMD to sample the full conformational landscape of peptoids. Thus, the “post-processing” aspect of BICePs can help to avoid the costly alternative of having to perform slow-to-converge simulations in the presence of restraints.

More recently, this approach was used to determine the solution-state structure and ion-binding mechanism of cyclic peptoid-spiroligomer hexamer macrocycles (Hurley et al., 2021). Northrup et al. found that particular sequences of alternating Q-proline and peptoid residues are able to bind metal cations, forming highly preorganized structures in the process (Northrup et al., 2020). To model this process, the BICePs algorithm was used to reconcile conformational populations from implicit-solvent REMD simulations in GAFF, against sparse experimental ROESY correlations. While GAFF simulations predict a range of macrocycle conformations with an overall preference for *cis*-amide backbones, the reweighted populations had a preference for *trans* amides, with the most populated conformation having five of six amides in the *trans* state. This conformation was then used to initiate more accurate explicit-solvent simulations of macrocycles in the presence of K^+^ and Na^+^ cations, in which several direct-binding events–coupled with a transition to an all-*trans* state–were observed. In validation of this model, the authors were able to correctly rank the ion-, solvent-, and sequence-dependence of cation-binding in agreement with experiment. Interestingly, a racemic crystal structure obtained for a peptoid-spiroligomer macrocycle in the absence of bound cation contains a mixture of *cis* and *trans* backbone amide, underscoring the need for an integrated modeling approach using BICePs to determine cation bound macrocycle conformations in solution.

### 3.3. Modeling Linear and Cyclic Peptides

Like peptoid foldamers, both cyclic and linear peptides can form preorganized structures in solution, and BICePs can be a valuable tool to help computationally model and design sequences with desirable solution-state properties. Wakefield et al. (2015) simulated 18 cyclic RGD peptides studied extensively by the Kessler group using NMR, including the anticancer drug candidate cilengitide, cyclo(RGDf-[*N*-Me]V), which targets integrin α_V_β_3_ (Dechantsreiter et al., 1999; Mas-Moruno et al., 2010). BICePs was used to validate excellent agreement between simulations and experimental NOE distances. The results reproduce the highly preorganized solution conformation of cilengitide, which has the highest affinity to integrin. Estimated differences in conformational entropy suggested that N-methylation provided about 0.5 kcal mol^−1^ of stabilization, and rigid non-natural amino acid mimics can provide even more preorganzation.

Ge and Voelz (2018) explored how the BICePs score could be used for force field validation and parameterization. Using a 2D lattice model as a toy system, they first demonstrated that BICePs was able to select the correct value of an interaction energy parameter given ensemble-averaged experimental distance measurements. The toy model was used to study the sensitivity of the results to the choice of reference potential, the number of conformational clusters used in the calculations, and the robustness of the calculation to experimental noise and measurement sparsity. In this work, the authors introduce support for chemical shift modeling in BICePs, which they use as experimental restraints to refine conformational populations of designed β-hairpin TrpLoop2 peptides in a number of force fields (Ge et al., 2017). BICePs results show unambiguously that explicit-solvent simulations in AMBER ff99-ildn-nmr (Li and Brüschweiler, 2010; Lindorff-Larsen et al., 2010) yield models most consistent with the experimental data. While this work suggests that BICePs is up to the task of model selection in the context of all-atom simulations, it also reveals several challenges that need to be overcome to perform these calculations reliably (see Discussion).

### 3.4. Reconciling Models of Globular Proteins With Experimental HDX Data

Recent work by Wan et al. expands the scope of BICePs—both in terms of system size and sampling complexity—by introducing support for yet another experimental observable: hydrogen/deuterium exchange (HDX) protection factors (Wan et al., 2020). HDX protection factors are challenging to enforce in molecular simulations, because they are *dynamical* restraints, corresponding to the relative rates of local unfolding events, where solvent exposure of backbone amides leads to exchange. For BICePs to refine structural ensembles using HDX protection factors, it requires a structural *proxy* that correlates with local unfolding, which the authors capture using the simple model:

(9) $$\ln{\text{PF}}_{i}=\beta_{c}{\langle N_{c}\rangle }_{i}+\beta_{h}{\langle N_{h}\rangle }_{i}+\beta_{0}.$$

In this model, the logarithm of the protection factor for residue *i* is predicted by the ensemble average number of heavy-atom contacts 〈_*N*_*c*_〉*i*_ and hydrogen bonds 〈_*N*_*h*_〉*i*_.

The free parameters in this model, λ (the β parameters and others defining how contacts and hydrogen bonds are tallied), are first determined using Bayesian inference, by training on two ultralong simulation trajectories of ubiquitin and bovine pancreatic trypsin inhibitor (BPTI), each well-studied systems with published experimental protection factors. The result is not a set of optimal (maximum-likelihood) parameters λ^*^, but rather the full posterior distribution of parameters *P*(λ), which is imported into the likelihood model for BICePs (More details can be found at https://github.com/vvoelz/HDX-forward-model). All parameters are then treated as nuisance parameters that are sampled in the BICePs posterior distribution.

To test this approach, Wan et al. applied the modified BICePs method to apomyoglobin, which has a disordered helix F and C-terminal portion of helix H in the absence of heme at pH 6. NMR studies provide no structural information for these regions, but HDX protection factors and chemical shifts are available. To model the structural ensemble of these regions, a series of simulations were performed at different temperatures and different bias potentials to encourage local unfolding. The resulting trajectory data was used to construct several competing multi-ensemble Markov Models (MEMMs) (Wu et al., 2016), where each could be evaluated using the BICePs score. The best-scoring model predicts a mixture of two predominant conformational states, one with a partially disordered yet compact helix F and other having a more disordered and exposed helix F, consistent with slow chemical exchange for helix F. Using the populations of these states predicted by BICePs, back-calculation of the HDX protection factors results in values that correlate well the experimental values (*R*^2^ = 0.72).

## 4. Discussion

In the future, we expect that BICePs will play an increasingly important role in molecular simulation-based prediction and design, for several reasons. First, unlike many similar algorithms for Bayesian inference, which enforce restraints during the course of a molecular simulation, BICePs can be implemented as a post-processing step. This means the algorithm should be considerably easier to implement and utilize across many applications.

Second, the ability to “tune” predictions of force fields using sparse experimental restraints can eliminate the need for custom parameterization, which can widen the scope of applications that can be addressed by general-purpose force fields. This is evidenced by the many examples of peptidomimetic and peptoid modeling we have described above. A further avenue, made possible by Markov state models (Prinz et al., 2011; Bowman et al., 2013), is to obtain reweighted predictions of equilibrium populations from BICePs to infer improved kinetic properties, through maximum caliber (MaxCal) approaches, for instance (Dixit et al., 2015; Wan et al., 2016; Ghosh et al., 2020).

Third, the BICePs score provides an unambiguous metric to rank model quality and perform model selection. As discussed above, this makes objective force field evaluation possible. Given a standard test set of molecular systems and associated corpus of experimental observables, BICePs could be a uniquely suitable Bayesian approach for systematically benchmarking and/or parameterizing new potentials. Similarly, the BICePs score could help quantify the progress toward an objective in adaptive sampling.

For BICePs to achieve the status of indispensable tool, there are several practical shortcomings and improvements that we are working to address.

### 4.1. Future Algorithmic Improvements

#### 4.1.1. Replica Averaging

One conceptual problem with BICePs and related methods like ISD is that the likelihood function compares individual conformational states to ensemble-averaged experimental observables. As result, the uncertainty parameter σ reflects a combination of both agreement with the experimental measurements and heterogeneity in the conformational ensemble (Bonomi et al., 2016a). A better comparison–and one that will result in lower uncertainty in most cases–is a likelihood function that compares a predicted ensemble-average to experimental observables. A simple way to achieve this, implemented currently in algorithms, such as Metainference (Löhr et al., 2019), is to use a forward model that incorporates the average of multiple MCMC *replicas*. In the limit of large numbers of replicas, such a likelihood function results in the least-biased, maximum entropy (MaxEnt) posterior distribution given ensemble-averaged experimental constraints (Pitera and Chodera, 2012; Cavalli et al., 2013; Roux and Weare, 2013; Hummer and Köfinger, 2015; Bonomi et al., 2016a; Xu, 2019).

One issue we believe replica averaging will improve is the performance of BICePs when used with many experimental restraints. This will increase the impact of BICePs by enabling its application to larger systems with many structural measurements. When modeling peptides with many NOE distance restraints (as in Ge et al., 2017; Ge and Voelz, 2018), we have noticed that while BICePs is able to correctly predict solution-state structures, it can overestimate the posterior populations of folded states. This occurs because particular conformational states that satisfy multiple restraints are highly rewarded by the likelihood function. This behavior is akin to the many constraint-based NMR structural refinement algorithms which seek to generate ensembles of structures that satisfy all or most distance constraints. A similar artifact was found by Ge et al. (2020) when evaluating MSM models of a series of cyclic β-hairpin peptides against structural NMR observables measured by Danelius et al. (2016).

In the replica-averaging section of the Discussion, we discuss this fairly extensively. The issue is not the system ^*^size^*^ *per se* (we have successfully applied BICePs to apomyglobin, a large globular protein, for example) but large numbers of experimental restraints, which become problematic because the likelihood function currently uses a forward model for individual states rather than ensemble-averages. In light of the reviewer's comments, we have added to this in our revised manuscript:

With replica averaging, direct comparison (via the BICePs score) between predictions from BICePs and constraint-based algorithms like NAMFIS (Cicero et al., 1995) should yield more favorable results.

#### 4.1.2. Hamiltonian Replica Exchange

As mentioned in the Theory section, better estimations of conformational populations and more accurate BICePs scores are achieved by implementing a free energy perturbation-like framework, in which parallel MCMC trajectories are perfomed for a series of theoretical priors scaled by λ ∈ [0, 1]. An issue that arises from this approach is the inability to sample all states in a reasonably low number of iterations, especially when λ = 1. To enhance the sampling of all the states (across all the λ-ensembles), we aim to implement Hamiltonian replica exchange in future versions of BICePs, an approach previously pioneered with ISD (Habeck et al., 2005). In this approach, parallel MCMC trajectories are coupled so that exchanges of conformational states across λ-ensembles are attempted at regular intervals and accepted according to the Metropolis criterion.

### 4.2. Support for More Experimental Observables and Reference Potentials

Another area of improvement we are working on is the incorporation of more experimental observables, and support for users to be able to extend BICePs by adding custom experimental restraints and reference potentials with relative ease. Our most recent addition to the roster of supported experimental observables is HDX protection factors, ln PF_*i*_. Custom experimental restraints will require a user to write a derived class and a few simple methods to parse input data files, compute a sum of squared errors, and specify the posterior−ln *P* (i.e., the energy function).

Small angle X-ray scattering (SAXS) has proven to be very useful for determining molecular shape and resolving structural dynamics over large range of biomolecular sizes (Bonomi et al., 2017). In the future, we hope to support SAXS observables as experimental restraints, joining the ranks of other Bayesian inference algorithms that can utilize such data (Antonov et al., 2016; Bonomi and Camilloni, 2017; Shevchuk and Hub, 2017; Potrzebowski et al., 2018). One issue to consider is how best to enforce uncertainties when mixed with other types of data, since SAXS experiments typically have a large number of not fully independent measurements. Here a Bayesian approach that can automatically “tune” uncertainties might be particularly powerful.

## 5. Conclusion

We have reviewed the theory and application of BICePs, an algorithm for Bayesian Inference of Conformational Populations, that has several advantages over similar methods. In BICePs, reweighting of populations can be performed as a post-processing step, with proper reference potentials. A review of previous applications demonstrates the utility of BICePs for improving the predictions of general-purpose force fields for modeling and designing peptidomimetics. A unique feature of the algorithm is the BICePs score, which can be used for objective, systematic model selection.

Since the first inception of the BICePs algorithm (Voelz and Zhou, 2014) (which we call “BICePs 1.0”) many modifications have been implemented, including support for more experimental observables, such as chemical shifts and HDX protection factors, and improved analysis and visualization. We have officially released the improved algorithm (BICePs 2.0) at https://github.com/vvoelz/biceps. This latest version is designed to lower the barriers for researchers to use and extended the BICePs algorithm.

## Author Contributions

VV, YG, and RR contributed to the conception, writing, and graphical figures in this work. All authors contributed to the article and approved the submitted version.

## Conflict of Interest

The authors declare that the research was conducted in the absence of any commercial or financial relationships that could be construed as a potential conflict of interest.
